# 4-(3-Methyl­anilino)-*N*-[*N*-(1-methyl­ethyl)carbamo­yl]pyridinium-3-sulfon­amidate (torasemide T–N): a low temperature redetermination

**DOI:** 10.1107/S1600536809012069

**Published:** 2009-04-08

**Authors:** Gianluca Bartolucci, Bruno Bruni, Silvia A. Coran, Massimo Di Vaira

**Affiliations:** a Dipartimento di Scienze Farmaceutiche, Universitá di Firenze, Via U. Schiff 6, I-50019 Sesto Fiorentino, Firenze, Italy; b Dipartimento di Chimica, Universitá di Firenze, Via della Lastruccia 3, I-50019 Sesto Fiorentino, Firenze, Italy

## Abstract

The structure [Danilovski *et al.* (2001 ▶). *Croat. Chim. Acta* **74**, 103–120] of the T–N (non-solvated) polymorph of torasemide, C_16_H_20_N_4_O_3_S, a diuretic drug used in the treatment of hypertension, has been redetermined at low temperature. The zwitterionic form of the mol­ecule is confirmed, although *GAUSSIAN03* calculations suggest that this form is less stable in the gas phase. The unit-cell contraction between 298 and 100 K is approximately isotropic and the largest structual change is in a C—N—C—C torsion angle, which differs by 11.4 (3)° between the room-temperature and low-temperature structures. There are two mol­ecules in the asymmetric unit, both of which contain an intra­molecular N—H⋯N hydrogen bond. In the crystal structure, both mol­ecules form inversion dimers linked by pairs of N—H⋯N hydrogen bonds. Further N—H⋯N and N—H⋯O hydrogen bonds lead to a three-dimensional network. The different hydrogen-bond arrangements and packing motifs in the polymorphs of torasemide are discussed in detail.

## Related literature

For the crystal structures of polymorphs of torasemide, see: Dupont *et al.* (1978 ▶); Danilovski *et al.* (2001 ▶). For the structure of the water–methanol solvated T–II form of torasemide, see: Bartolucci *et al.* (2009 ▶).
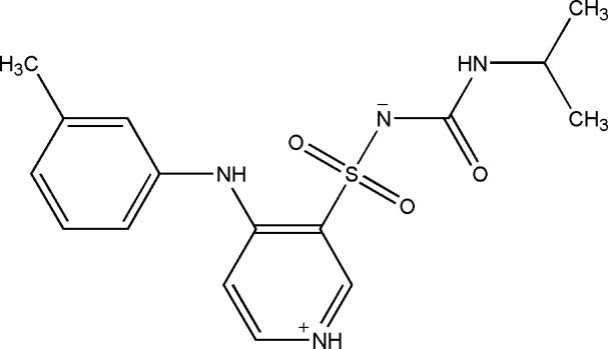


## Experimental

### 

#### Crystal data

C_16_H_20_N_4_O_3_S
*M* *_r_* = 348.42
Monoclinic, 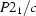

*a* = 11.3378 (1) Å
*b* = 18.9055 (1) Å
*c* = 16.4958 (1) Å
β = 94.273 (1)°
*V* = 3525.99 (4) Å^3^
*Z* = 8
Cu *K*α radiation
μ = 1.82 mm^−1^
*T* = 100 K
0.50 × 0.20 × 0.15 mm

#### Data collection

Oxford Diffraction Xcalibur PX Ultra CCD diffractometer
Absorption correction: multi-scan (ABSPACK; Oxford Diffraction, 2006 ▶) *T*_min_ = 0.541, *T*_max_ = 1.000 (expected range = 0.412–0.761)
53231 measured reflections
6941 independent reflections
6840 reflections with *I* > 2σ(*I*)
*R*_int_ = 0.029

#### Refinement

*R*[*F* ^2^ > 2σ(*F* ^2^)] = 0.043
*wR*(*F* ^2^) = 0.117
*S* = 1.07
6941 reflections
458 parameters
H atoms treated by a mixture of independent and constrained refinement
Δρ_max_ = 0.64 e Å^−3^
Δρ_min_ = −0.36 e Å^−3^

### 

Data collection: *CrysAlisPro CCD* (Oxford Diffraction, 2006 ▶); cell refinement: *CrysAlisPro CCD*; data reduction: *CrysAlisPro RED* (Oxford Diffraction, 2006 ▶); program(s) used to solve structure: *SIR97* (Altomare *et al.*, 1999 ▶); program(s) used to refine structure: *SHELXL97* (Sheldrick, 2008 ▶); molecular graphics: *ORTEP-3* (Farrugia, 1997 ▶); software used to prepare material for publication: *SHELXL97*, *WinGX* (Farrugia, 1999 ▶), *GAUSSIAN03* (Frisch *et al.*, 2004 ▶) and *PARST* (Nardelli, 1995 ▶).

## Supplementary Material

Crystal structure: contains datablocks global, I. DOI: 10.1107/S1600536809012069/hb2935sup1.cif

Structure factors: contains datablocks I. DOI: 10.1107/S1600536809012069/hb2935Isup2.hkl

Additional supplementary materials:  crystallographic information; 3D view; checkCIF report

## Figures and Tables

**Table 1:** Hydrogen-bond geometry (Å, °)

*D*—H⋯*A*	*D*—H	H⋯*A*	*D*⋯*A*	*D*—H⋯*A*
N1—H1*N*⋯N3	0.87 (2)	2.50 (2)	3.0897 (18)	126.1 (17)
N5—H5*N*⋯N7	0.91 (2)	2.21 (2)	2.9399 (19)	136.4 (18)
N4—H4*N*⋯O1^i^	0.89 (2)	2.16 (2)	3.0400 (18)	175 (2)
N8—H8*N*⋯O5^ii^	0.90 (2)	2.01 (2)	2.9067 (18)	175 (2)
N1—H1*N*⋯N3^i^	0.87 (2)	2.20 (2)	2.8977 (19)	137.4 (18)
N5—H5*N*⋯N7^ii^	0.91 (2)	2.37 (2)	3.0591 (19)	132.9 (17)
N2—H2*N*⋯O4	0.93 (2)	2.33 (2)	2.8674 (18)	116.8 (16)
N6—H6*N*⋯O2^iii^	0.85 (2)	2.08 (2)	2.8184 (18)	145 (2)
N2—H2*N*⋯O6	0.93 (2)	1.77 (2)	2.6286 (17)	153.4 (19)
N6—H6*N*⋯O3^iii^	0.85 (2)	2.37 (2)	2.9660 (19)	128.2 (18)

Symmetry codes: (i) 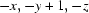; (ii) 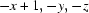; (iii) 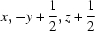.

**Fig. 1.**
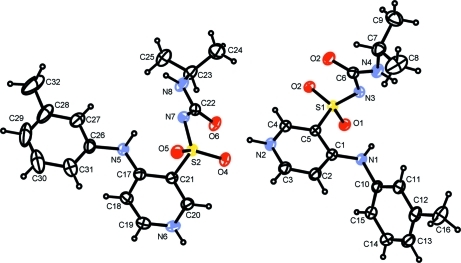
A view of the content of the asymmetric unit of (I). Displacement ellipsoids are drawn at the 50% probability level.

**Fig. 2.**
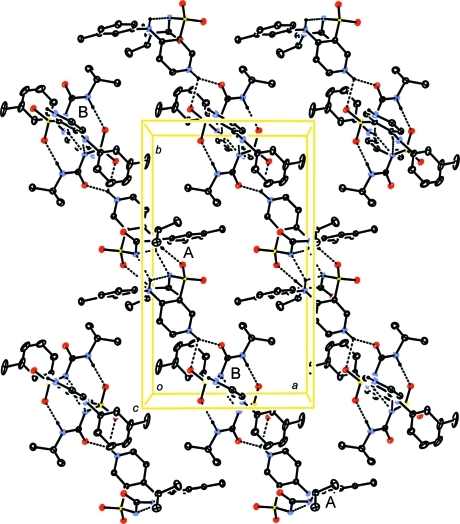
A view of the crystal packing in the structure of (I), in proximity of the ab face. Hydrogen bonds are denoted by dashed lines. Only hydrogen atoms involved in the formation of hydrogen bonds are shown. The A and B labels denote centrosymmetric molecule dimers, respectively formed by the symmetry–independent molecules of the two types, present in the structure. The dimers, joined by hydrogen bonds, form chains parallel to the [1–10] direction (or to the [110] direction, on parallel planes at c/2 distance from the one shown).

**Fig. 3.**
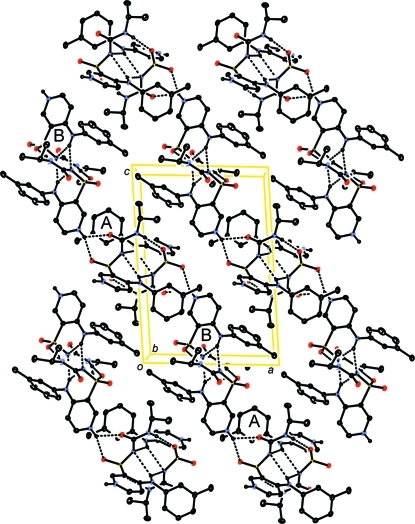
The arrangement of chains formed by hydrogen–bonded molecule dimers lying in proximity of the ac face. The same type of arrangement, with all chains consistently aligned in the [10–1] direction, exists on the neighbouring parallel planes, at b/2 distance from the one shown.

## supplementary crystallographic information

### Comment

Three polymorphs of torasemide have been reported up to now, respectively denoted T–I, T–II (Dupont *et al.*, 1978) and T–N (Danilovski *et al.*, 2001). In addition, the structure of a water–methanol solvate, isomorphous with form T–II has now been determined (Bartolucci *et al.*, 2009). Since crystals of the T–N form were repeatedly obtained in the course of an investigation on torasemide polymorphic forms, it was deemed worthwhile to collect a set of low–temperature (100 K) data on this structure (I), in order to enable comparisons with the results of the previous, accurate, room–temperature study of the same T–N form. Also, it appeared that a new, unified, approach to the description of hydrogen bonding in the T–N, T–II and T–II solvate structures would be useful. The asymmetric unit of the monoclinic unit cell of I (Fig. 1) contains two symmetry–independent molecules of torasemide (as for form T–II; however, no solvent molecules are present in the T–N structure). As already pointed out by Danilovski *et al.*, the T–N form has the highest density among all known polymorphs. Moreover, there is a 3% decrease in the cell volume going from room temperature to 100 K, the decrease being rather isotropic, possibly due to the rather uniform, three–dimensional, distribution of hydrogen–bond linkages (hydrogen bonds and the effects on their distances due to the decrease in temperature are considered below). As far as the molecular conformation is concerned, only the most flexible parts are significantly affected by the temperature decrease, with a 11.4 (3)° change in the value of the C6–N4–C7–C8 torsion angle and a 4.1° variation for the C22–N8–C23–C24 one (labelling criteria are consistent with those of the accompanying paper on the T–II solvated form: the molecules formed by carbon atoms C1 to C16 and C17 to C32 respectively correspond to the A and B molecules in Danilovski's notation). Other conformational changes are smaller, the largest one, 3.2 (3)°, being found for the torsion angle of the S1–N3–C6–N4 chain. The values of the angles between the best planes through the aromatic rings of the two molecules, 81.60 (5)° (A) and 63.21 (6)° (B), are close to those found in the room–temperature study [80.3 (2)° and 62.8 (3)°, respectively]. The N–H amine bonds are oriented as in the structure of the T–II solvate and the large difference (*ca* 0.09 Å) between the lengths of the two N–C bonds formed by N1 and, separately, by N5, discussed in connection with the T–II solvate structure, is found also for the T–N polymorph.

The three–dimensional network of hydrogen bonds (the order of entries in Table 1 corresponds to that of the report of the room–temperature investigation) presents strong similarities with that of the T–II form, although these are elusive, due to differences in the reference systems. As in the T–II structure, there are centrosymmetric dimers of molecule A, internally connnected by the N1···N3 (N1···N3 = 3.090 (2) Å, N1—H1N···N3 = 126 (2)°), N1···N3^i^ (N1···N3^i^ = 2.898 (2) Å, N1—H1N···N3^i^ = 137 (2)°; symmetry code (i): - *x*, 1 - *y*, - *z*) and N4···O1^i^ (N4···O1^i^ = 3.040 (2) Å, N4—H4N···O1^i^ = 175 (2)°) hydrogen bonds and centrosymmetric dimers of molecule B, linked by the N5···N7 (N5···N7 = 2.940 (2) Å, N5—H5N···N7 = 136 (2)°), N5···N7^ii^ (N5···N7^ii^ = 3.059 (2) Å, N5—H5N···N7^ii^ = 133 (2)°; symmetry code (ii): 1 - *x*, - *y*, - *z*) and N8···O5^ii^ (N8···O5^ii^ = 2.907 (2) Å, N8—H8N···O5^ii^ = 175 (2)°) hydrogen bonds. In a similar way to the arrangement of the T–II polymorph, dimers of the above two types are connected through bifurcated hydrogen bonds, namely, N2···O4 (N2···O4 = 2.867 (2) Å, N2—H2N···O4 = 117 (2)°) and N2···O6 (N2···O6 = 2.629 (2) Å, N2—H2N···O6 = 153 (2)°), forming chains characterized by the AABBAA sequence of molecules. These chains are stacked sideways forming planes parallel to the *ab* cell face (Fig. 2). On adjacent planes of this set, spaced by *c*/2 intervals, the chains are alternatively parallel to the [1–10] and [110] directions. As in the structure of the T—II form, a three–dimensional network of hydrogen bonds is attained through connections between molecule dimers belonging to adjacent planes of the former set. The latter links involve the N6···O2^iii^ (N6···O2^iii^ = 2.818 (2) Å, N6—H6N···O2^iii^ = 145 (2)°; symmetry code (iii): *x*, 1/2 - *y*, 1/2 + *z*) and N6···O3^iii^ (N6···O3^iii^ = 2.966 (2) Å, N6—H6N···O3^iii^ = 128 (2)°) hydrogen bonds, in such a way that a distinct set of planar arrays of AABBAA chains is generated, these planes being parallel to the *ac* cell face (Fig. 3). At variance with the arrangement existing in the T–II structure (but consistently with the difference between the space groups of the T–II and T–N forms), the chains on *all* planes of the latter set have the same [10–1] orientation. With the exceptions of the intramolecular N1···N3 and N5···N7 hydrogen bonds, the lengths of all the other hydrogen bonds decrease with decreasing temperature, the largest effects (*ca* 0.08 Å decrease from room temperature to 100 K) occurring for the intradimer intermolecular N1···N3^i^ and N5···N7^ii^ linkages.

Since all recent torasemide structure determinations have unambiguously shown that the molecule adopts the zwitterionic form, it was interesting to compare the energy of this arrangement with that of the tautomer where the N3, or N7, nitrogen is protonated, instead of the pyridine nitrogen. Geometry optimizations performed with the *GAUSSIAN03* programs suite at the B3LYP/6–31 G(d,p) level, followed by single–point calculations on the optimized geometries, using the 6–311++G(d,p) basis set, yielded the zwitterionic form as definitely less stable than the other one (by as much as 56.6 kJ/mol) in the gas phase. However, the energy gap reduces to 5.3 kJ/mol if the presence of a dielectric environment is simulated by the PCM model, suggesting that the zwitterionic form is actually stabilized by the hydrogen bond interactions existing in water solution as well as in the solid state. Both optimized geometries showed the appreciable difference in the lengths of the two N–C bonds formed by the amine N1, or N5, atom (this point, recalled above, is discussed in the accompanying paper).

### Experimental

Samples of torasemide were kindly provided by SIMS (SIMS srl, Reggello Firenze, Italy). Crystals of (I), suitable for X-ray diffraction analysis, were obtained by slow evaporation from methanol solutions.

### Refinement

H atoms bound to carbon atoms were in geometrically generated positions, riding, whereas the coordinates of those bound to the N atoms were refined freely. The constraint *U(H)* = 1.2*U*_eq_(C,*N*) on hydrogen temperature factors was applied [*U(H)* = 1.5*U*_eq_(C) for the H atoms of methyl groups]. The N—H bond distances formed by refined hydrogen atoms were in the range 0.85 – 0.93 Å.

### Crystal data

**Table:**

C_16_H_20_N_4_O_3_S	*F*(000) = 1472
*M**_r_* = 348.42	*D*_x_ = 1.313 Mg m^−^^3^
Monoclinic, *P*2_1_/*c*	Cu *K*α radiation, λ = 1.5418 Å
Hall symbol: -P 2ybc	Cell parameters from 43504 reflections
*a* = 11.3378 (1) Å	θ = 4.6–72.2°
*b* = 18.9055 (1) Å	µ = 1.82 mm^−^^1^
*c* = 16.4958 (1) Å	*T* = 100 K
β = 94.273 (1)°	Prism, colorless
*V* = 3525.99 (4) Å^3^	0.50 × 0.20 × 0.15 mm
*Z* = 8	

### Data collection

**Table:**

Oxford Diffraction Xcalibur PX Ultra CCD diffractometer	6941 independent reflections
Radiation source: fine-focus sealed tube	6840 reflections with *I* > 2σ(*I*)
Oxford Diffraction, Enhance ULTRA assembly	*R*_int_ = 0.029
Detector resolution: 8.1241 pixels mm^-1^	θ_max_ = 72.6°, θ_min_ = 4.6°
ω scans	*h* = −13→13
Absorption correction: multi-scan (ABSPACK; Oxford Diffraction, 2006)	*k* = −19→23
*T*_min_ = 0.541, *T*_max_ = 1.000	*l* = −20→17
53231 measured reflections	

### Refinement

**Table:**

Refinement on *F*^2^	Secondary atom site location: difference Fourier map
Least-squares matrix: full	Hydrogen site location: inferred from neighbouring sites
*R*[*F*^2^ > 2σ(*F*^2^)] = 0.043	H atoms treated by a mixture of independent and constrained refinement
*wR*(*F*^2^) = 0.117	*w* = 1/[σ^2^(*F*_o_^2^) + (0.0678*P*)^2^ + 1.9103*P*] where *P* = (*F*_o_^2^ + 2*F*_c_^2^)/3
*S* = 1.07	(Δ/σ)_max_ = 0.001
6941 reflections	Δρ_max_ = 0.64 e Å^−^^3^
458 parameters	Δρ_min_ = −0.36 e Å^−^^3^
0 restraints	Extinction correction: *SHELXL97* (Sheldrick, 2008), Fc^*^=kFc[1+0.001xFc^2^λ^3^/sin(2θ)]^-1/4^
Primary atom site location: structure-invariant direct methods	Extinction coefficient: 0.0029 (2)

### Special details

**Table:**

Geometry. All e.s.d.'s (except the e.s.d. in the dihedral angle between two l.s. planes) are estimated using the full covariance matrix. The cell e.s.d.'s are taken into account individually in the estimation of e.s.d.'s in distances, angles and torsion angles; correlations between e.s.d.'s in cell parameters are only used when they are defined by crystal symmetry. An approximate (isotropic) treatment of cell e.s.d.'s is used for estimating e.s.d.'s involving l.s. planes.
Refinement. Refinement of *F*^2^ against ALL reflections. The weighted *R*-factor *wR* and goodness of fit *S* are based on *F*^2^, conventional *R*-factors *R* are based on *F*, with *F* set to zero for negative *F*^2^. The threshold expression of *F*^2^ > σ(*F*^2^) is used only for calculating *R*-factors(gt) *etc*. and is not relevant to the choice of reflections for refinement. *R*-factors based on *F*^2^ are statistically about twice as large as those based on *F*, and *R*- factors based on ALL data will be even larger.

### Fractional atomic coordinates and isotropic or equivalent isotropic displacement parameters (Å^2^)

**Table:**

	*x*	*y*	*z*	*U*_iso_*/*U*_eq_	
C1	0.06628 (14)	0.35277 (8)	0.09441 (9)	0.0240 (3)	
C2	0.05152 (15)	0.28496 (9)	0.12963 (10)	0.0292 (3)	
H2	−0.0181	0.2749	0.1562	0.035*	
C3	0.13599 (15)	0.23451 (9)	0.12564 (11)	0.0308 (4)	
H3	0.1238	0.1893	0.1487	0.037*	
N2	0.23689 (12)	0.24714 (7)	0.08976 (9)	0.0281 (3)	
H2N	0.2975 (19)	0.2144 (11)	0.0880 (13)	0.034*	
C4	0.25680 (14)	0.31069 (8)	0.05726 (9)	0.0253 (3)	
H4	0.3287	0.3186	0.0326	0.030*	
C5	0.17588 (13)	0.36451 (8)	0.05876 (9)	0.0232 (3)	
S1	0.20832 (3)	0.446859 (19)	0.01391 (2)	0.02230 (12)	
O1	0.19638 (10)	0.49999 (6)	0.07600 (7)	0.0259 (2)	
O2	0.32755 (10)	0.44000 (6)	−0.01170 (7)	0.0259 (2)	
N3	0.10577 (12)	0.45781 (7)	−0.05325 (8)	0.0242 (3)	
C6	0.09635 (14)	0.41075 (8)	−0.11840 (9)	0.0253 (3)	
O3	0.17766 (10)	0.37454 (6)	−0.14317 (7)	0.0285 (3)	
N4	−0.01530 (13)	0.40839 (8)	−0.15352 (9)	0.0319 (3)	
H4N	−0.069 (2)	0.4363 (12)	−0.1341 (14)	0.038*	
C7	−0.04833 (16)	0.36875 (10)	−0.22750 (10)	0.0330 (4)	
H7	0.0095	0.3292	−0.2316	0.040*	
C8	−0.1707 (2)	0.33682 (15)	−0.22238 (16)	0.0605 (7)	
H81	−0.1694	0.3039	−0.1764	0.091*	
H82	−0.1936	0.3113	−0.2728	0.091*	
H83	−0.2279	0.3746	−0.2146	0.091*	
C9	−0.0425 (2)	0.41555 (12)	−0.30182 (12)	0.0474 (5)	
H91	0.0377	0.4345	−0.3035	0.071*	
H92	−0.0987	0.4547	−0.2989	0.071*	
H93	−0.0627	0.3877	−0.3510	0.071*	
N1	−0.01871 (12)	0.40159 (7)	0.09416 (8)	0.0252 (3)	
H1N	−0.0117 (18)	0.4407 (12)	0.0675 (13)	0.030*	
C10	−0.11809 (14)	0.39528 (8)	0.14235 (10)	0.0247 (3)	
C11	−0.22992 (14)	0.38155 (8)	0.10547 (10)	0.0279 (3)	
H11	−0.2402	0.3758	0.0482	0.033*	
C12	−0.32729 (15)	0.37622 (9)	0.15230 (11)	0.0306 (4)	
C13	−0.30887 (15)	0.38601 (9)	0.23606 (11)	0.0327 (4)	
H13	−0.3740	0.3823	0.2689	0.039*	
C14	−0.19782 (16)	0.40100 (10)	0.27239 (11)	0.0338 (4)	
H14	−0.1876	0.4084	0.3294	0.041*	
C15	−0.10120 (15)	0.40520 (9)	0.22556 (10)	0.0300 (3)	
H15	−0.0245	0.4148	0.2503	0.036*	
C16	−0.44868 (16)	0.36000 (11)	0.11375 (14)	0.0426 (5)	
H161	−0.4641	0.3092	0.1178	0.064*	
H162	−0.4532	0.3739	0.0564	0.064*	
H163	−0.5079	0.3863	0.1419	0.064*	
C17	0.53049 (14)	0.00721 (8)	0.20469 (10)	0.0255 (3)	
C18	0.57906 (15)	0.00421 (9)	0.28673 (10)	0.0287 (3)	
H18	0.6541	−0.0171	0.2990	0.034*	
C19	0.51868 (16)	0.03167 (9)	0.34779 (10)	0.0303 (3)	
H19	0.5517	0.0286	0.4023	0.036*	
N6	0.41244 (13)	0.06327 (8)	0.33205 (9)	0.0295 (3)	
H6N	0.3709 (19)	0.0776 (12)	0.3695 (14)	0.035*	
C20	0.36491 (15)	0.07020 (9)	0.25530 (10)	0.0267 (3)	
H20	0.2912	0.0938	0.2455	0.032*	
C21	0.42078 (14)	0.04394 (8)	0.19141 (10)	0.0246 (3)	
S2	0.34526 (3)	0.048572 (19)	0.09286 (2)	0.02303 (12)	
O4	0.24685 (10)	0.09590 (6)	0.10138 (7)	0.0273 (2)	
O5	0.31276 (10)	−0.02365 (6)	0.07219 (7)	0.0267 (2)	
N7	0.43996 (12)	0.07155 (7)	0.03378 (8)	0.0241 (3)	
C22	0.48491 (14)	0.13952 (8)	0.03857 (9)	0.0247 (3)	
O6	0.44602 (10)	0.18959 (6)	0.07821 (7)	0.0297 (3)	
N8	0.57991 (14)	0.14797 (8)	−0.00463 (10)	0.0335 (3)	
H8N	0.611 (2)	0.1105 (12)	−0.0284 (14)	0.040*	
C23	0.64106 (16)	0.21554 (9)	−0.00994 (12)	0.0337 (4)	
H23	0.6350	0.2413	0.0425	0.040*	
C24	0.5836 (2)	0.26095 (11)	−0.07816 (14)	0.0468 (5)	
H241	0.5935	0.2382	−0.1306	0.070*	
H242	0.6211	0.3077	−0.0771	0.070*	
H243	0.4991	0.2662	−0.0707	0.070*	
C25	0.77101 (18)	0.20277 (11)	−0.02102 (15)	0.0462 (5)	
H251	0.8055	0.1739	0.0241	0.069*	
H252	0.8125	0.2482	−0.0218	0.069*	
H253	0.7790	0.1779	−0.0725	0.069*	
N5	0.58380 (12)	−0.02241 (8)	0.14337 (8)	0.0278 (3)	
H5N	0.5523 (18)	−0.0126 (11)	0.0923 (13)	0.033*	
C26	0.67680 (15)	−0.07370 (10)	0.15013 (10)	0.0303 (4)	
C27	0.77484 (16)	−0.06222 (12)	0.10718 (11)	0.0383 (4)	
H27	0.7847	−0.0182	0.0808	0.046*	
C28	0.86000 (18)	−0.11553 (17)	0.10247 (13)	0.0596 (7)	
C29	0.8433 (3)	−0.17915 (16)	0.14258 (17)	0.0703 (9)	
H29	0.9001	−0.2158	0.1398	0.084*	
C30	0.7461 (3)	−0.18997 (14)	0.18613 (17)	0.0644 (7)	
H30	0.7368	−0.2336	0.2135	0.077*	
C31	0.66150 (19)	−0.13720 (11)	0.19027 (13)	0.0449 (5)	
H31	0.5941	−0.1445	0.2202	0.054*	
C32	0.9641 (2)	−0.1025 (2)	0.05306 (16)	0.0944 (14)	
H321	1.0160	−0.1440	0.0560	0.142*	
H322	1.0082	−0.0611	0.0746	0.142*	
H323	0.9361	−0.0938	−0.0037	0.142*	

### Atomic displacement parameters (Å^2^)

**Table:**

	*U*^11^	*U*^22^	*U*^33^	*U*^12^	*U*^13^	*U*^23^
C1	0.0236 (7)	0.0249 (8)	0.0240 (7)	0.0036 (6)	0.0044 (6)	0.0007 (6)
C2	0.0273 (8)	0.0261 (8)	0.0355 (9)	0.0035 (6)	0.0113 (6)	0.0048 (7)
C3	0.0320 (9)	0.0259 (8)	0.0359 (9)	0.0042 (6)	0.0122 (7)	0.0056 (7)
N2	0.0283 (7)	0.0238 (7)	0.0333 (7)	0.0073 (5)	0.0089 (6)	0.0037 (5)
C4	0.0239 (7)	0.0269 (8)	0.0256 (7)	0.0032 (6)	0.0050 (6)	0.0012 (6)
C5	0.0218 (7)	0.0261 (7)	0.0221 (7)	0.0033 (6)	0.0044 (6)	0.0014 (6)
S1	0.0216 (2)	0.0232 (2)	0.0225 (2)	0.00266 (13)	0.00451 (14)	0.00186 (13)
O1	0.0261 (6)	0.0259 (5)	0.0259 (5)	0.0018 (4)	0.0036 (4)	−0.0026 (4)
O2	0.0214 (5)	0.0303 (6)	0.0266 (6)	0.0024 (4)	0.0063 (4)	0.0031 (4)
N3	0.0247 (7)	0.0253 (6)	0.0229 (6)	0.0046 (5)	0.0030 (5)	0.0020 (5)
C6	0.0271 (8)	0.0247 (7)	0.0245 (7)	0.0027 (6)	0.0057 (6)	0.0033 (6)
O3	0.0264 (6)	0.0274 (6)	0.0326 (6)	0.0037 (4)	0.0083 (5)	−0.0015 (5)
N4	0.0269 (7)	0.0400 (8)	0.0285 (7)	0.0082 (6)	0.0003 (6)	−0.0081 (6)
C7	0.0321 (9)	0.0366 (9)	0.0305 (8)	0.0023 (7)	0.0022 (7)	−0.0072 (7)
C8	0.0474 (13)	0.0741 (17)	0.0611 (14)	−0.0202 (12)	0.0124 (11)	−0.0272 (13)
C9	0.0630 (13)	0.0464 (12)	0.0317 (10)	0.0050 (10)	−0.0029 (9)	−0.0017 (8)
N1	0.0226 (6)	0.0245 (7)	0.0295 (7)	0.0049 (5)	0.0084 (5)	0.0059 (5)
C10	0.0230 (7)	0.0219 (7)	0.0297 (8)	0.0043 (6)	0.0063 (6)	0.0020 (6)
C11	0.0263 (8)	0.0242 (8)	0.0333 (8)	0.0030 (6)	0.0040 (6)	−0.0036 (6)
C12	0.0248 (8)	0.0231 (8)	0.0447 (10)	0.0011 (6)	0.0076 (7)	−0.0053 (7)
C13	0.0287 (8)	0.0307 (8)	0.0405 (9)	0.0026 (7)	0.0142 (7)	0.0015 (7)
C14	0.0339 (9)	0.0391 (9)	0.0294 (8)	0.0062 (7)	0.0083 (7)	0.0017 (7)
C15	0.0251 (8)	0.0345 (9)	0.0306 (8)	0.0044 (6)	0.0038 (6)	0.0015 (7)
C16	0.0264 (9)	0.0416 (10)	0.0606 (12)	−0.0022 (7)	0.0077 (8)	−0.0177 (9)
C17	0.0261 (8)	0.0252 (8)	0.0256 (8)	0.0003 (6)	0.0053 (6)	0.0020 (6)
C18	0.0290 (8)	0.0304 (8)	0.0263 (8)	−0.0007 (6)	−0.0002 (6)	0.0033 (6)
C19	0.0357 (9)	0.0318 (8)	0.0233 (8)	−0.0049 (7)	0.0016 (6)	0.0017 (6)
N6	0.0338 (8)	0.0320 (7)	0.0236 (7)	−0.0029 (6)	0.0088 (6)	−0.0025 (6)
C20	0.0287 (8)	0.0260 (8)	0.0261 (8)	−0.0001 (6)	0.0058 (6)	−0.0013 (6)
C21	0.0270 (8)	0.0238 (7)	0.0236 (7)	0.0017 (6)	0.0050 (6)	0.0009 (6)
S2	0.0245 (2)	0.0222 (2)	0.0226 (2)	0.00471 (13)	0.00328 (14)	−0.00076 (13)
O4	0.0248 (6)	0.0261 (6)	0.0314 (6)	0.0077 (4)	0.0044 (4)	−0.0015 (4)
O5	0.0298 (6)	0.0226 (5)	0.0282 (6)	0.0024 (4)	0.0047 (4)	−0.0023 (4)
N7	0.0280 (7)	0.0227 (6)	0.0221 (6)	0.0047 (5)	0.0055 (5)	0.0001 (5)
C22	0.0259 (7)	0.0247 (7)	0.0236 (7)	0.0052 (6)	0.0024 (6)	0.0006 (6)
O6	0.0260 (6)	0.0259 (6)	0.0379 (6)	0.0040 (4)	0.0077 (5)	−0.0065 (5)
N8	0.0347 (8)	0.0243 (7)	0.0438 (8)	0.0011 (6)	0.0176 (6)	−0.0062 (6)
C23	0.0319 (9)	0.0294 (9)	0.0413 (9)	−0.0001 (7)	0.0121 (7)	−0.0068 (7)
C24	0.0486 (12)	0.0396 (11)	0.0533 (12)	−0.0040 (9)	0.0116 (9)	0.0056 (9)
C25	0.0343 (10)	0.0426 (11)	0.0636 (13)	−0.0012 (8)	0.0152 (9)	−0.0112 (10)
N5	0.0269 (7)	0.0334 (7)	0.0231 (7)	0.0092 (6)	0.0030 (5)	0.0018 (5)
C26	0.0269 (8)	0.0358 (9)	0.0278 (8)	0.0099 (7)	−0.0024 (6)	−0.0032 (7)
C27	0.0264 (9)	0.0600 (12)	0.0278 (9)	0.0069 (8)	−0.0019 (7)	−0.0106 (8)
C28	0.0317 (10)	0.107 (2)	0.0382 (11)	0.0312 (12)	−0.0102 (8)	−0.0284 (12)
C29	0.0653 (17)	0.0767 (18)	0.0643 (15)	0.0491 (15)	−0.0254 (13)	−0.0237 (14)
C30	0.0738 (17)	0.0438 (13)	0.0712 (16)	0.0247 (12)	−0.0234 (14)	−0.0020 (11)
C31	0.0448 (11)	0.0383 (10)	0.0502 (12)	0.0078 (8)	−0.0062 (9)	0.0068 (9)
C32	0.0323 (12)	0.200 (4)	0.0498 (14)	0.0384 (18)	−0.0021 (10)	−0.037 (2)

### Geometric parameters (Å, °)

**Table:**

C1—N1	1.334 (2)	C17—N5	1.339 (2)
C1—C2	1.422 (2)	C17—C18	1.424 (2)
C1—C5	1.431 (2)	C17—C21	1.427 (2)
C2—C3	1.357 (2)	C18—C19	1.362 (2)
C2—H2	0.9500	C18—H18	0.9500
C3—N2	1.348 (2)	C19—N6	1.352 (2)
C3—H3	0.9500	C19—H19	0.9500
N2—C4	1.342 (2)	N6—C20	1.345 (2)
N2—H2N	0.93 (2)	N6—H6N	0.85 (2)
C4—C5	1.372 (2)	C20—C21	1.363 (2)
C4—H4	0.9500	C20—H20	0.9500
C5—S1	1.7740 (16)	C21—S2	1.7823 (16)
S1—O1	1.4481 (11)	S2—O4	1.4452 (11)
S1—O2	1.4518 (11)	S2—O5	1.4483 (12)
S1—N3	1.5589 (13)	S2—N7	1.5643 (13)
N3—C6	1.393 (2)	N7—C22	1.382 (2)
C6—O3	1.2416 (19)	C22—O6	1.2495 (19)
C6—N4	1.353 (2)	C22—N8	1.345 (2)
N4—C7	1.457 (2)	N8—C23	1.459 (2)
N4—H4N	0.89 (2)	N8—H8N	0.90 (2)
C7—C9	1.517 (3)	C23—C25	1.517 (2)
C7—C8	1.521 (3)	C23—C24	1.523 (3)
C7—H7	1.0000	C23—H23	1.0000
C8—H81	0.9800	C24—H241	0.9800
C8—H82	0.9800	C24—H242	0.9800
C8—H83	0.9800	C24—H243	0.9800
C9—H91	0.9800	C25—H251	0.9800
C9—H92	0.9800	C25—H252	0.9800
C9—H93	0.9800	C25—H253	0.9800
N1—C10	1.4316 (19)	N5—C26	1.431 (2)
N1—H1N	0.87 (2)	N5—H5N	0.91 (2)
C10—C15	1.384 (2)	C26—C27	1.379 (3)
C10—C11	1.389 (2)	C26—C31	1.388 (3)
C11—C12	1.397 (2)	C27—C28	1.402 (3)
C11—H11	0.9500	C27—H27	0.9500
C12—C13	1.394 (3)	C28—C29	1.393 (4)
C12—C16	1.504 (2)	C28—C32	1.504 (4)
C13—C14	1.383 (3)	C29—C30	1.375 (4)
C13—H13	0.9500	C29—H29	0.9500
C14—C15	1.389 (2)	C30—C31	1.389 (3)
C14—H14	0.9500	C30—H30	0.9500
C15—H15	0.9500	C31—H31	0.9500
C16—H161	0.9800	C32—H321	0.9800
C16—H162	0.9800	C32—H322	0.9800
C16—H163	0.9800	C32—H323	0.9800
			
N1—C1—C2	121.19 (14)	N5—C17—C18	122.48 (15)
N1—C1—C5	122.72 (14)	N5—C17—C21	121.66 (14)
C2—C1—C5	116.09 (14)	C18—C17—C21	115.86 (14)
C3—C2—C1	120.58 (15)	C19—C18—C17	120.50 (15)
C3—C2—H2	119.7	C19—C18—H18	119.7
C1—C2—H2	119.7	C17—C18—H18	119.7
N2—C3—C2	121.47 (15)	N6—C19—C18	121.06 (15)
N2—C3—H3	119.3	N6—C19—H19	119.5
C2—C3—H3	119.3	C18—C19—H19	119.5
C4—N2—C3	120.51 (14)	C20—N6—C19	120.83 (15)
C4—N2—H2N	116.0 (13)	C20—N6—H6N	116.7 (15)
C3—N2—H2N	123.4 (13)	C19—N6—H6N	122.4 (15)
N2—C4—C5	121.63 (15)	N6—C20—C21	121.05 (15)
N2—C4—H4	119.2	N6—C20—H20	119.5
C5—C4—H4	119.2	C21—C20—H20	119.5
C4—C5—C1	119.66 (14)	C20—C21—C17	120.57 (15)
C4—C5—S1	118.93 (12)	C20—C21—S2	117.91 (12)
C1—C5—S1	121.39 (11)	C17—C21—S2	121.19 (12)
O1—S1—O2	113.94 (7)	O4—S2—O5	114.95 (7)
O1—S1—N3	107.85 (7)	O4—S2—N7	117.35 (7)
O2—S1—N3	117.80 (7)	O5—S2—N7	106.83 (7)
O1—S1—C5	106.29 (7)	O4—S2—C21	105.28 (7)
O2—S1—C5	105.64 (7)	O5—S2—C21	105.36 (7)
N3—S1—C5	104.25 (7)	N7—S2—C21	106.05 (7)
C6—N3—S1	118.27 (11)	C22—N7—S2	119.33 (11)
O3—C6—N4	122.33 (15)	O6—C22—N8	121.01 (15)
O3—C6—N3	126.15 (15)	O6—C22—N7	126.24 (15)
N4—C6—N3	111.52 (14)	N8—C22—N7	112.75 (14)
C6—N4—C7	123.33 (14)	C22—N8—C23	122.60 (14)
C6—N4—H4N	118.5 (15)	C22—N8—H8N	119.8 (14)
C7—N4—H4N	117.8 (15)	C23—N8—H8N	117.4 (14)
N4—C7—C9	110.66 (16)	N8—C23—C25	109.75 (15)
N4—C7—C8	109.64 (15)	N8—C23—C24	111.02 (16)
C9—C7—C8	111.79 (19)	C25—C23—C24	111.51 (17)
N4—C7—H7	108.2	N8—C23—H23	108.1
C9—C7—H7	108.2	C25—C23—H23	108.1
C8—C7—H7	108.2	C24—C23—H23	108.1
C7—C8—H81	109.5	C23—C24—H241	109.5
C7—C8—H82	109.5	C23—C24—H242	109.5
H81—C8—H82	109.5	H241—C24—H242	109.5
C7—C8—H83	109.5	C23—C24—H243	109.5
H81—C8—H83	109.5	H241—C24—H243	109.5
H82—C8—H83	109.5	H242—C24—H243	109.5
C7—C9—H91	109.5	C23—C25—H251	109.5
C7—C9—H92	109.5	C23—C25—H252	109.5
H91—C9—H92	109.5	H251—C25—H252	109.5
C7—C9—H93	109.5	C23—C25—H253	109.5
H91—C9—H93	109.5	H251—C25—H253	109.5
H92—C9—H93	109.5	H252—C25—H253	109.5
C1—N1—C10	122.61 (13)	C17—N5—C26	126.59 (14)
C1—N1—H1N	119.9 (14)	C17—N5—H5N	116.5 (13)
C10—N1—H1N	117.3 (14)	C26—N5—H5N	116.5 (13)
C15—C10—C11	120.92 (15)	C27—C26—C31	121.01 (17)
C15—C10—N1	118.87 (14)	C27—C26—N5	118.10 (16)
C11—C10—N1	120.19 (14)	C31—C26—N5	120.38 (17)
C10—C11—C12	120.28 (16)	C26—C27—C28	120.0 (2)
C10—C11—H11	119.9	C26—C27—H27	120.0
C12—C11—H11	119.9	C28—C27—H27	120.0
C13—C12—C11	118.17 (16)	C29—C28—C27	118.4 (2)
C13—C12—C16	120.72 (16)	C29—C28—C32	122.7 (3)
C11—C12—C16	121.11 (17)	C27—C28—C32	118.9 (3)
C14—C13—C12	121.40 (15)	C30—C29—C28	121.3 (2)
C14—C13—H13	119.3	C30—C29—H29	119.3
C12—C13—H13	119.3	C28—C29—H29	119.3
C13—C14—C15	120.07 (16)	C29—C30—C31	120.1 (3)
C13—C14—H14	120.0	C29—C30—H30	119.9
C15—C14—H14	120.0	C31—C30—H30	119.9
C10—C15—C14	119.15 (16)	C26—C31—C30	119.1 (2)
C10—C15—H15	120.4	C26—C31—H31	120.4
C14—C15—H15	120.4	C30—C31—H31	120.4
C12—C16—H161	109.5	C28—C32—H321	109.5
C12—C16—H162	109.5	C28—C32—H322	109.5
H161—C16—H162	109.5	H321—C32—H322	109.5
C12—C16—H163	109.5	C28—C32—H323	109.5
H161—C16—H163	109.5	H321—C32—H323	109.5
H162—C16—H163	109.5	H322—C32—H323	109.5
			
N1—C1—C2—C3	176.46 (16)	N5—C17—C18—C19	−176.36 (16)
C5—C1—C2—C3	−2.6 (2)	C21—C17—C18—C19	3.7 (2)
C1—C2—C3—N2	1.2 (3)	C17—C18—C19—N6	−1.0 (3)
C2—C3—N2—C4	0.4 (3)	C18—C19—N6—C20	−1.9 (3)
C3—N2—C4—C5	−0.4 (2)	C19—N6—C20—C21	1.8 (2)
N2—C4—C5—C1	−1.2 (2)	N6—C20—C21—C17	1.1 (2)
N2—C4—C5—S1	−179.61 (12)	N6—C20—C21—S2	174.53 (12)
N1—C1—C5—C4	−176.47 (15)	N5—C17—C21—C20	176.28 (16)
C2—C1—C5—C4	2.6 (2)	C18—C17—C21—C20	−3.7 (2)
N1—C1—C5—S1	1.9 (2)	N5—C17—C21—S2	3.1 (2)
C2—C1—C5—S1	−178.98 (12)	C18—C17—C21—S2	−176.91 (12)
C4—C5—S1—O1	−125.64 (13)	C20—C21—S2—O4	12.97 (15)
C1—C5—S1—O1	55.93 (14)	C17—C21—S2—O4	−173.67 (13)
C4—C5—S1—O2	−4.25 (15)	C20—C21—S2—O5	−108.94 (13)
C1—C5—S1—O2	177.32 (12)	C17—C21—S2—O5	64.42 (14)
C4—C5—S1—N3	120.55 (13)	C20—C21—S2—N7	138.01 (13)
C1—C5—S1—N3	−57.88 (14)	C17—C21—S2—N7	−48.63 (15)
O1—S1—N3—C6	−177.62 (11)	O4—S2—N7—C22	47.61 (14)
O2—S1—N3—C6	51.70 (14)	O5—S2—N7—C22	178.36 (11)
C5—S1—N3—C6	−64.93 (13)	C21—S2—N7—C22	−69.62 (13)
S1—N3—C6—O3	−23.6 (2)	S2—N7—C22—O6	−10.9 (2)
S1—N3—C6—N4	156.37 (12)	S2—N7—C22—N8	168.51 (12)
O3—C6—N4—C7	−5.9 (3)	O6—C22—N8—C23	−1.7 (3)
N3—C6—N4—C7	174.09 (15)	N7—C22—N8—C23	178.84 (15)
C6—N4—C7—C9	−92.7 (2)	C22—N8—C23—C25	149.93 (18)
C6—N4—C7—C8	143.5 (2)	C22—N8—C23—C24	−86.3 (2)
C2—C1—N1—C10	12.9 (2)	C18—C17—N5—C26	15.8 (3)
C5—C1—N1—C10	−168.03 (15)	C21—C17—N5—C26	−164.23 (16)
C1—N1—C10—C15	73.0 (2)	C17—N5—C26—C27	−131.35 (18)
C1—N1—C10—C11	−108.95 (18)	C17—N5—C26—C31	56.8 (3)
C15—C10—C11—C12	−1.2 (2)	C31—C26—C27—C28	1.0 (3)
N1—C10—C11—C12	−179.23 (14)	N5—C26—C27—C28	−170.78 (16)
C10—C11—C12—C13	0.9 (2)	C26—C27—C28—C29	−0.6 (3)
C10—C11—C12—C16	−178.81 (16)	C26—C27—C28—C32	178.09 (19)
C11—C12—C13—C14	0.4 (3)	C27—C28—C29—C30	−0.2 (3)
C16—C12—C13—C14	−179.96 (17)	C32—C28—C29—C30	−178.8 (2)
C12—C13—C14—C15	−1.3 (3)	C28—C29—C30—C31	0.6 (4)
C11—C10—C15—C14	0.2 (2)	C27—C26—C31—C30	−0.6 (3)
N1—C10—C15—C14	178.32 (15)	N5—C26—C31—C30	171.00 (19)
C13—C14—C15—C10	1.0 (3)	C29—C30—C31—C26	−0.2 (3)

### Hydrogen-bond geometry (Å, °)

**Table:**

*D*—H···*A*	*D*—H	H···*A*	*D*···*A*	*D*—H···*A*
N1—H1N···N3	0.87 (2)	2.50 (2)	3.0897 (18)	126.1 (17)
N5—H5N···N7	0.91 (2)	2.21 (2)	2.9399 (19)	136.4 (18)
N4—H4N···O1^i^	0.89 (2)	2.16 (2)	3.0400 (18)	175 (2)
N8—H8N···O5^ii^	0.90 (2)	2.01 (2)	2.9067 (18)	175 (2)
N1—H1N···N3^i^	0.87 (2)	2.20 (2)	2.8977 (19)	137.4 (18)
N5—H5N···N7^ii^	0.91 (2)	2.37 (2)	3.0591 (19)	132.9 (17)
N2—H2N···O4	0.93 (2)	2.33 (2)	2.8674 (18)	116.8 (16)
N6—H6N···O2^iii^	0.85 (2)	2.08 (2)	2.8184 (18)	145 (2)
N2—H2N···O6	0.93 (2)	1.77 (2)	2.6286 (17)	153.4 (19)
N6—H6N···O3^iii^	0.85 (2)	2.37 (2)	2.9660 (19)	128.2 (18)

Symmetry codes: (i) −*x*, −*y*+1, −*z*; (ii) −*x*+1, −*y*, −*z*; (iii) *x*, −*y*+1/2, *z*+1/2.
